# Safety and Efficacy of Vitamin K Antagonists vs. Novel Oral Anticoagulants in Patients With Left Ventricular Thrombus: A Meta-Analysis

**DOI:** 10.3389/fcvm.2021.636491

**Published:** 2021-04-29

**Authors:** He Xuan, Yi-Ming Chen, Yun-Lang Dai, Jing Zhou, Yu-Feng Jiang, Ya-Feng Zhou

**Affiliations:** ^1^Department of Cardiology, Dushu Lake Hospital Affiliated to Soochow University, Suzhou, China; ^2^Department of Cardiology, The First Affiliated Hospital of Soochow University, Suzhou, China

**Keywords:** novel oral anticoagulant, vitamin K antagonist, bleeding, thromboembolic events, left ventricular thrombus

## Abstract

**Aims:** A meta-analysis was conducted to evaluate the safety and efficacy of novel oral anticoagulants (NOACs) compared with vitamin K antagonists (VKAs) in patients with left ventricular thrombus (LVT).

**Methods and Results:** We searched PubMed, Web of Science, and Cochrane Library for cohort studies comparing the use of VKAs vs. NOACs for the treatment of LVT from the earliest date available to September 30, 2020. The predetermined efficacy and safety outcomes included thromboembolic events, resolution of LVT, clinically significant bleedings, and all-cause death. Fixed-effects model was used to estimate the pooled effects. Publication bias analyses and sensitivity analyses were conducted to check the robustness of results. A total of 6 studies enrolling 837 patients (mean age 60.2 ± 1.6 years; 77.2% were male) were included. We found no significant differences in thromboembolic events [relative risk (RR) 1.69, 95% confidence interval (CI) 0.94–3.06, *P* 0.08, I^2^ 12.7%], the rate of resolution of thrombus (RR 1.08, 95% CI 0.96–1.21, *P* 0.21, I^2^ 4.8%), and clinically significant bleedings (RR 0.70, 95% CI 0.37–1.32, *P* 0.27, I^2^ 0%) between the VKAs and NOACs group. Additionally, no significant difference in all-cause mortality was found between the two groups (RR 1.24, 95% CI 0.79–1.96, *P* 0.35, I^2^ 0.0%). Sensitivity analyses, using the “1-study removed” method, detected no significant differences.

**Conclusion:** NOACs and VKAs have similar efficacy and safety in treating LVT, prompting the inference that NOACs are the possible alternatives of VKAs in LVT therapy.

## Introduction

Left ventricular thrombus (LVT) is a common complication of acute myocardial infarction (MI) (1, 2), and is also observed in patients with non-ischemic cardiomyopathies with severe left ventricular systolic dysfunction (3). Previous studies have suggested that LVT can significantly increase the risk of developing embolic events by 5.5-fold (4, 5). Current guidelines recommend the use of vitamin K antagonists (VKAs) in patients with post-MI LVT (6, 7). However, as its slow onset and the fluctuation of drug concentration, regular monitoring of international normalized ratio (INR) and constant adjustment for warfarin dosage are required to achieve a safe and efficacious outcome, which may potentially lead to decreased patient compliance (8).

Novel oral anticoagulants (NOACs) are the first-line treatments against thromboembolic events in patients with non-valvular atrial fibrillation (9). Considering the efficacy and safety of NOACs, NOACs are sometimes used off-label for anticoagulation therapy in patients with LVT in clinical practice. To date, most studies of NOACs for the management of thrombotic events in patients with LVT are case reports and cohort studies with limited sample sizes.

So far, several studies have been conducted to compare the safety and efficacy of VKAs vs. NOACs for anticoagulation therapy in patients with LVT. Recently, a meta-analysis performed by Cochran et al. showed non-inferiority of NOACs in treating LVT compared to VKAs (10); however, half of the studies included were abstracts without full manuscript, which may hamper the generalization of the result. Moreover, several studies have been published after the time of Cochran's meta-analysis (11–13). Hence, we conducted the meta-analysis, which included only studies published in peer-reviewed journals, to evaluate the safety and efficacy of NOACs compared with VKAs in patients with LVT.

## Methods

### Search Strategy and Selection Criteria

We systemically searched PubMed, Web of Science, and Cochrane Library database for relevant studies published before September 30, 2020. Search items included “Factor Xa inhibitor”, “direct oral anticoagulant”, “NOAC”, “DOAC”, “dabigatran”, “rivaroxaban”, “edoxaban”, “apixaban” combined with “vitamin K antagonist”, “Warfarin” and “left ventricular thrombi^*^”. We also checked the reference lists of obtained articles to avoid omissions. Abstracts, meeting proceedings, and private communications were not included in this study.

The articles included should meet the following inclusion criteria: (1) published studies in English in peer-reviewed journals; (2) adult patients diagnosed with LVT; (3) data about the efficacy and safety in LVT patients taking VKAs or NOACs was available. For studies with overlapping cohorts, the article with the most comprehensive data would be included for analysis.

We followed the Preferred Reporting Items for Systematic Reviews and Meta-Analysis (PRISMA) guidelines (14) and the Cochrane Handbook for Systematic Reviews to perform and report this systematic review and meta-analysis.

### Data Extraction and Quality Assessment

YC and HX independently screened all retrieved articles and discussed it with a third investigator (YD) when facing disagreements. Inclusion and exclusion criteria, baseline characteristics of the included patients, treatment methods, and all outcomes in each group were extracted. We extracted the most comprehensively adjusted or unadjusted hazard ratios (HRs) and 95% confidence intervals (CIs) from Cox proportional hazards analysis. Otherwise, we extracted an exact number of specific outcomes.

We used the Newcastle-Ottawa Quality Assessment Scale for Cohort Studies (NOS) (15) to assess the bias of each study. According to NOS, each study would get 0~4 points in selecting of the study groups, 0~2 points in the comparability of the study group, and 0~3 points in the ascertainment of the outcome of interest. Then studies were classified into high (total 0~4 points), medium (total 5~7 points), and low (total 8~9 points) risk of bias.

### Outcomes Assessment

The efficacy endpoints were thromboembolic events, consisting of stroke, transient ischemic attack, and peripheral artery embolism during the period of observation, and resolution of LVT (defined as no evidence of thrombus on repeat imaging). Safety endpoints were clinically significant bleedings (defined as in the individual article) and all-cause mortality.

### Data Analysis

We used relative risks (RRs) with the 95% CIs to compare the differences between VKAs and NOACs group for the meta-analyses. Q test was used to evaluate the heterogeneity of included studies with I^2^ and *P*-values. The pooled RRs were calculated by the fixed-effects model according to the Mantel-Haenszel method or by the random-effects model with Der Simonian and Laird method for studies with present heterogeneity (I^2^ > 50% or *P*-value < 0.05). We detected the publication bias of articles by drawing the funnel plot with the Egger test. Furthermore, the contour-funnel plot in conjunction with the trim-and-fill method was used to identify the causes of asymmetry observed in a funnel plot (16). In addition, we conducted sensitivity analyses with the “1-study removed” method to check the credibility of the results. All data analysis was carried out using Stata/SE 15.1.

## Results

### Results of the Literature Search

**Figure 1:**
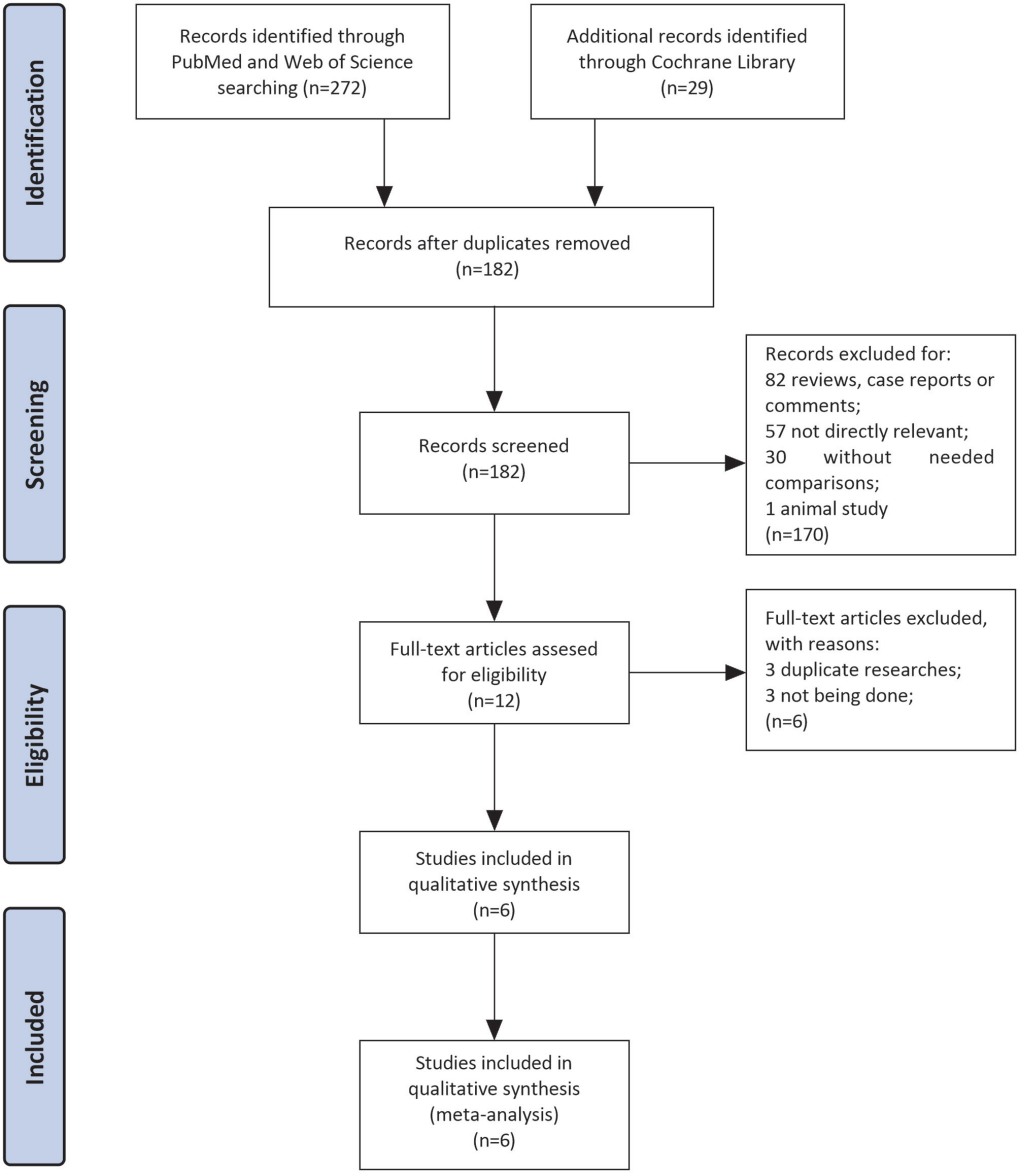
The flowchart for complete screening process.

Three hundred and one studies were enrolled after searching three databases mentioned above. After the removal of duplicates, there were remaining 182 articles. Eighty-two articles were excluded because they were reviews, case reports, or letters. In the left 100 articles, 57 studies were excluded for not investigating patients with LVT, 30 studies for without comparison between VKAs and NOACs, and 1 for animal study. Finally, we performed full-text screening on the remaining 12 documents: 3 of them were excluded for not being done (17–19), and 3 were duplicate researches (20–22). Then six studies left for subsequent data analyses with sample sizes ranged from 59 to 421 (10–13, 23, 24). The complete screening process was shown as a flow chart in Figure 1.

### Study Characteristics and Quality

The patient characteristics were shown in Table 1. A total of 837 patients in 6 studies were included in this meta-analysis. Overall, 77.2% were male, and the mean age of patients was 60.2 ± 1.6 years old. Transthoracic echocardiography (TTE) was the primary method for the diagnosis of LVT, and cardiac magnetic resonance imaging (CMR) was also performed in some patients to make the diagnosis. Ischemic cardiomyopathy (67.9%) dominated in the etiology of LVT. Of the 234 (28.0%) patients who received NOACs, rivaroxaban (43/99, 43.4%) and apixaban (50/99, 50.5%) were preferred NOACs; however, details of anticoagulant regimens were not reported in the remaining 135 subjects (10, 24). The doses of NOACs chosen in the included studies were identical to those applied in the primary stroke prevention for patients with atrial fibrillation. All included studies were of low to medium bias, and details of NOS assessment can be found in Table 1.

**Table 1:** Baseline characteristics of included studies.

**Study**	**Country**	**Design**	**Sample size (NOAC/VKA/n)**	**Mean age (years)**	**Male (%)**	**LVEF**	**ICM**	**Follow up (months)**	**NOACs used**	**Newcastle-Ottawa Scale**		
						**(%)**	**(%)**			**Selection**	**Comparability**	**Outcome**
Iqbal et al. (12)	UK	retrospective	22/62/84	62 ± 14	89.3	34 ± 13	86.9	36 ± 16.8	Apixaban; Dabigatran, Rivaroxaban	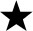 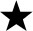 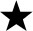 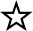	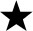 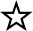	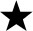 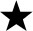 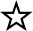
Jones et al. (11)	UK	prospective	41/60/101	59.6 ± 14.1	85.1	34.5 ± 9.6	100	median 26.4	Apixaban, Edoxaban, Rivaroxaban	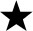 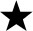 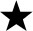 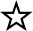	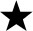 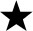	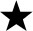 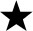 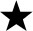
Guddeti et al. (13)	USA	retrospective	19/80/99	61 ± 12.3	71	25	58.6	10.4 ± 3.4	Apixaban; Dabigatran; Rivaroxaban.	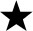 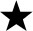 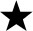 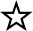	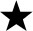 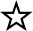	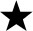 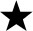 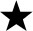
Daher et al. (23)	France	retrospective	17/42/59	62 ± 14	83.1	37 ± 11	86.5	NR	Apixaban, Dabigatran, Rivaroxaban	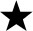 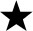 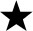 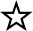	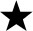 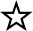	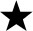 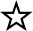 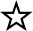
Cochran et al. (10)	USA	retrospective	14/59/73	NR	76.7	NR	58.9	12	Apixaban, Dabigatran, Rivaroxaban, Edoxaban	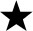 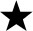 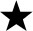 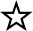	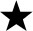 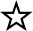	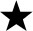 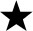 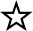
Robinson et al. (24)	USA	retrospective	121/236/421^*^	58.4 ± 14.8	73.7	27.6	59.4	median 11.7	Apixaban, Dabigatran, Rivaroxaban	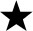 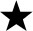 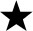 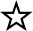	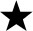 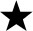	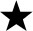 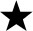 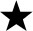

*ICM, ischemic cardiomyopathy; LVEF, left ventricular ejection fraction; NOAC, novel oral anticoagulants; VKA, vitamin K antagonists*.

* *including a mixed cohort of 64 patients who switched the treatment. NR refers to no available data*.

### Efficacy Endpoints

**Figure 2:**
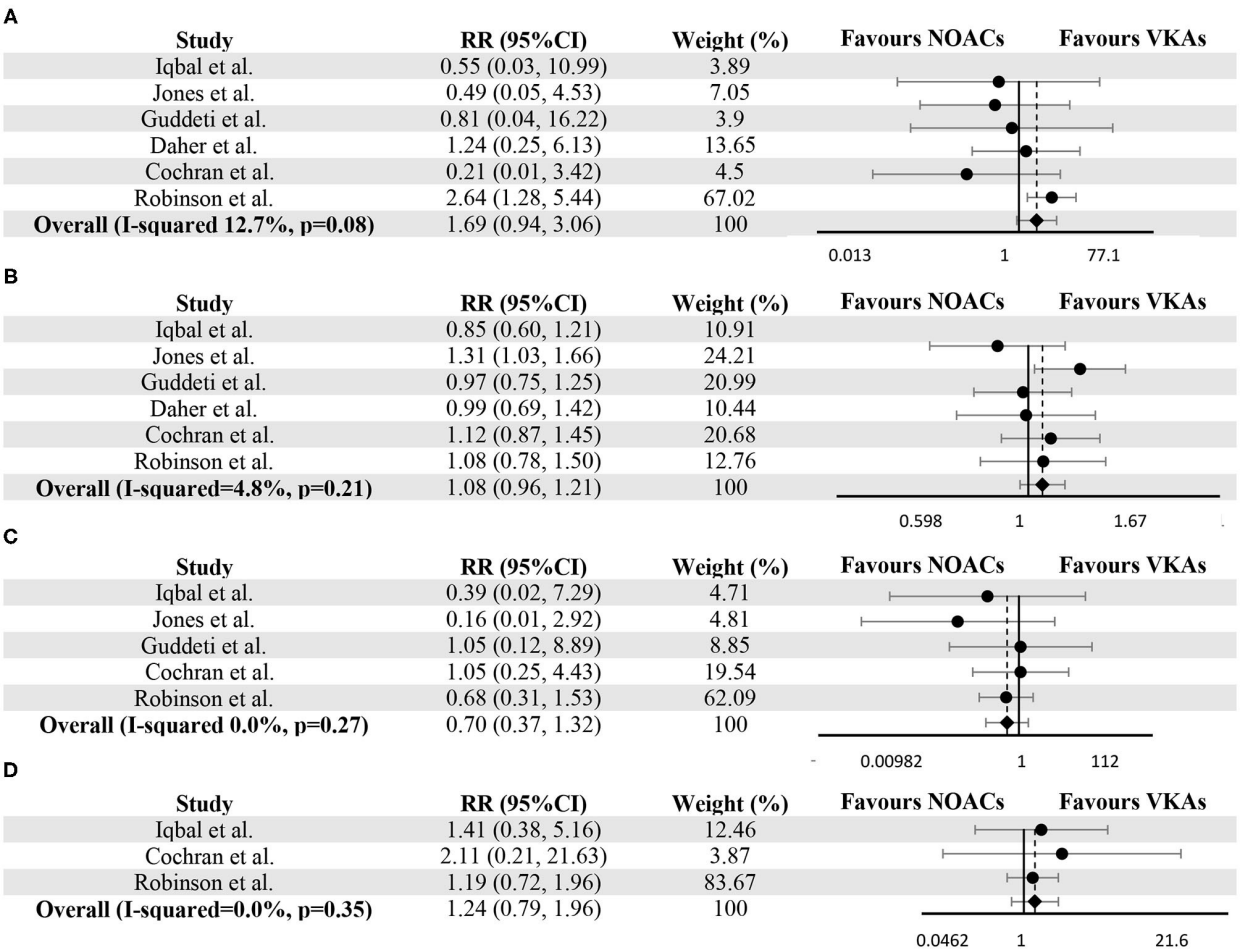
Meta-analysis of vitamin K antagonists vs. novel oral anticoagulants for the endpoints of thromboembolic events **(A)**, resolution of LVT **(B)**, clinically significant bleedings **(C)**, and all-cause death **(D)**. Tests for differences were based on *T*-tests using fixed effect models. RR, risk ratio; CI, confidence interval.

The resolution of LVT and thromboembolic events were reported in all six studies. During the follow-up, thromboembolic events and resolution of LVT occurred in 54 (6.5%) and 483 (58.3%) subjects, respectively. No difference was detected between the NOACs group and the VKAs group (RR 1.08, 95% CI 0.96–1.21, *P* 0.21, I^2^ 4.8%) for the resolution of LVT. As for thromboembolic events, there was no significant difference between the two groups (RR 1.69, 95% CI 0.94–3.06, *P* 0.08, I^2^ 12.7%), and this conclusion did not change after excluding 64 individuals who switched treatment (RR 1.35, 95% CI 0.72–2.51, *P* 0.35, I^2^ 0.0%) (Supplementary Figure 1). Forest plots for comparisons of resolution of LVT and thromboembolic events between two groups were shown in Figure 2.

### Safety Outcomes

In LVT therapy, clinically significant bleedings were considered as the essential parameters for evaluating safety. Five studies provided the number of bleeding events in both groups (10–13, 24). The occurrence of clinically significant bleedings (as defined in the individual study in Supplementary Table 1) was not significantly different between VKAs and NOACs group (RR 0.70, 95% CI 0.37–1.32, *P* 0.27, I^2^ 0.0%) (Figure 2C). As for all-cause death, Iqbal et al. (12), Cochran et al. (10) and Robinson et al. (24) provided the relevant data, and no significant difference can be detected (RR 1.24, 95% CI 0.79–1.96, *P* 0.35, I^2^ 0.0%) (Figure 2D).

### Publication Bias

**Figure 3:**
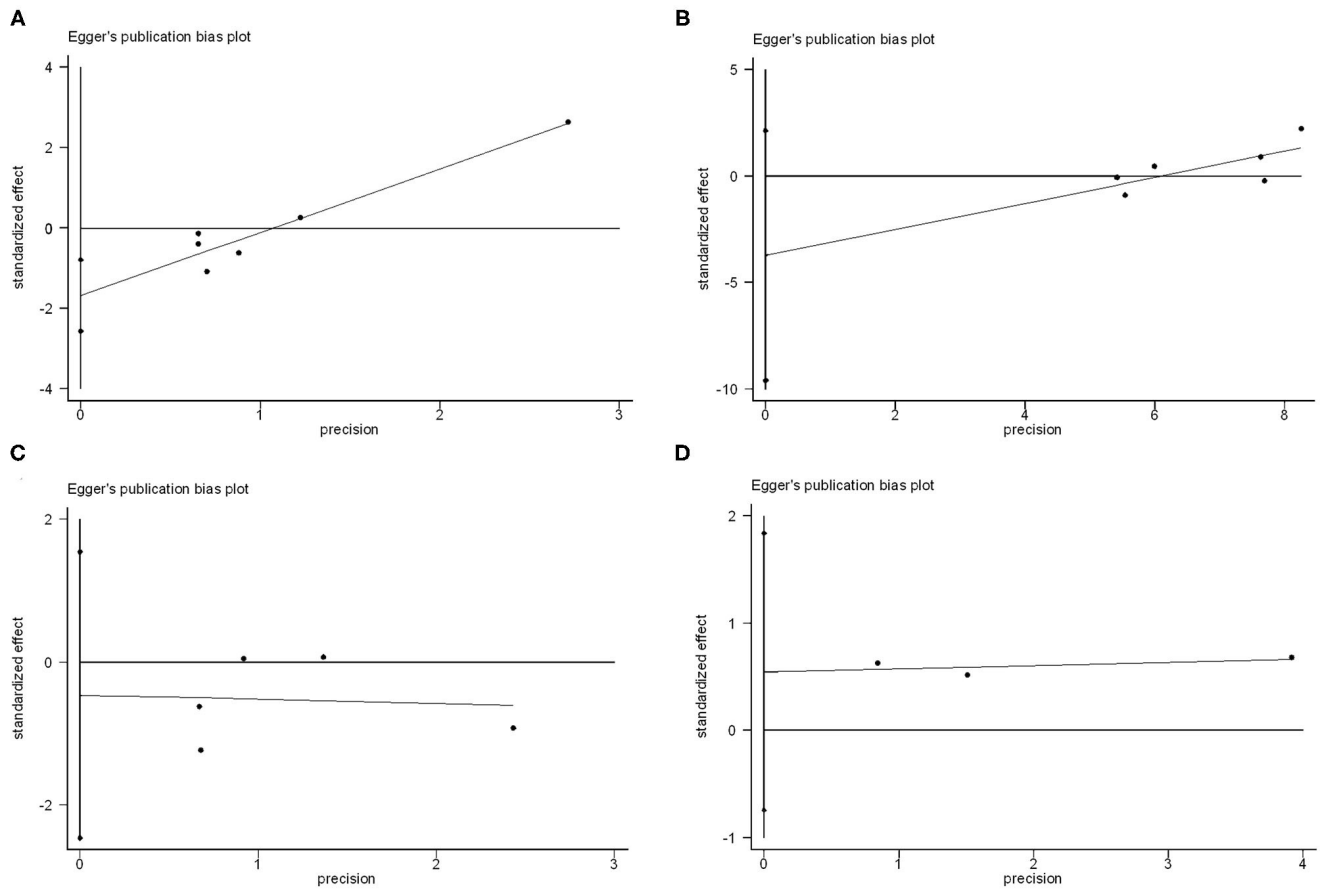
Publication bias analysis of vitamin K antagonists vs. novel oral anticoagulants for the endpoints of thromboembolic events **(A)**, resolution of LVT **(B)**, clinically significant bleedings **(C)**, and all-cause death **(D)** by Egger's test.

Funnel plots for individual endpoint were drawn, and no asymmetry was found when evaluated with the Egger's test except for thromboembolic events (P for thromboembolic events = 0.01, P for bleeding = 0.52, P for resolution of thrombosis = 0.15, P for death = 0.12) (Figure 3). However, the contour-funnel plot in conjunction with the trim-and-fill method implied the observed asymmetry in the funnel plot for thromboembolic events might not be due to publication bias, as no “missing” studies were indicated in the Supplementary Figure 2.

### Sensitivity Analyses

**Figure 4:**
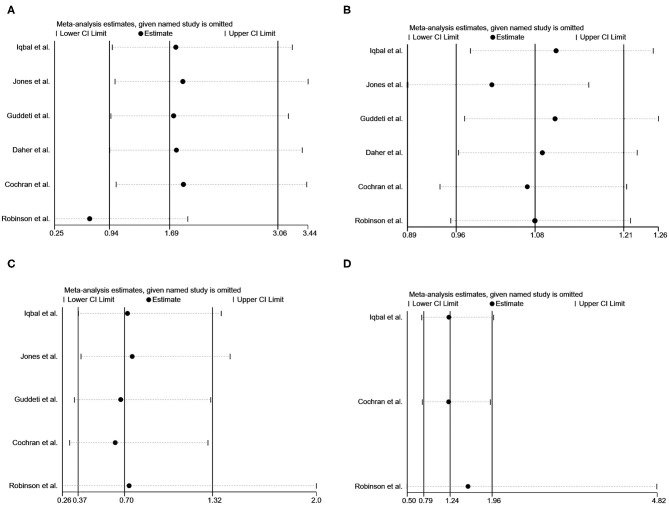
Sensitivity analysis of vitamin K antagonists vs. novel oral anticoagulants for the endpoints of thromboembolic events **(A)**, resolution of LVT **(B)**, clinically significant bleedings **(C)**, and all-cause death **(D)** with the “1-study removed” method.

Sensitivity analyses by sequentially removing a single study were performed, detecting no significant difference except for pooling the data of thromboembolic events after removing the study by Robinson et al. (24) (Figure 4).

## Discussion

LVT is a severe complication of cardiovascular diseases, most commonly occurred in patients with anterior myocardial infarction. The overall incidence of developing LVT in patients with acute MI is about 17% in the thrombolytic era and has decreased to 3% with the universal access to percutaneous coronary intervention (PCI) (25). Nevertheless, the incidence of LVT in patients with anterior MI is still high (9%) (26). Besides, LVT is closely related to several adverse cardiovascular events during the 1-year follow-up (27).

According to the American College of Cardiology Foundation/American Heart Association Guidelines, patients with ST-segment elevation myocardial infarction (STEMI) and LVT are recommended to receive anticoagulant therapy with VKAs for 3 months, similar to suggestions in the AHA/American Stroke Association 2014 guidelines on stroke prevention (6, 7). While the European Society of Cardiology 2017 STEMI guidelines suggest the use of anticoagulants without specific recommendations on strategies to prevent LVT (28). Periodic monitoring of TTE is recommended in all three guidelines.

VKAs block the enzyme vitamin K epoxide reductase, thereby inhibiting reactivation of vitamin K1, which is essential to synthesize coagulation factors II, VII, IX, and X. Besides, VKAs can also inhibit anticoagulant proteins C and protein S but to a lesser extent. Warfarin is the most commonly used VKA. However, apart from its slow onset and the fluctuation of drug concentration, warfarin is known to interact with many common drugs and certain foods (29). Thus, there are well-recognized difficulties for the management of anticoagulation with VKAs.

NOACs are highly selective Xa factor or direct thrombin inhibitors. Due to the specificity of reversely combining with the Xa factor, NOACs can be administrated without the need for routine coagulation monitoring, which could potentially increase patient compliance (30). Although NOACs are less effective in the prevention of systematic thromboembolic events in patients with mechanic valves (31), the long-term use of NOACs as the first-line anticoagulants in patients with left atrial thrombus and non-valvular atrial fibrillation has been demonstrated to have satisfying efficacy and safety (32). However, due to the intrinsic mechanistic differences between LVT and left atrial thrombus [the former may involve both the blood stasis and endocardial damage (33)], clinical evidence of anticoagulant use derived from studies based on patients with atrial fibrillation may not be applicable to patients with LVT (31).

Recently, some studies have noted that NOACs may have similar efficacy to warfarin in the treatment of LVT but reached opposite conclusions. The study by Robinson et al. showed the superiority of VKAs over NOACs for the thromboembolic events; nevertheless, the superiority disappeared when the analysis was restricted to patients who did not switch treatment (RR 1.99, 95% CI 0.91–4.35, *P* 0.08) or based on intention-to-treatment analysis (RR 1.42, 95% CI 0.68–1.92, *P* 0.35) (24). In this meta-analysis, after pooling 837 patients of LVT treated with VKAs or NOACs from six studies, no difference was found in the resolution of LVT (RR 1.08, 95% CI 0.96–1.21, *P* 0.212, I^2^ 4.8%) and thromboembolic events (RR 1.69, 95% CI 0.94–3.06, *P* 0.08, I^2^ 12.7%) between VKAs and NOACs group, indicating the similar efficacy of VKAs and NOACs in LVT therapy. As for analyses of clinically significant bleedings and death, no significant differences were detected. The heterogeneity tests for the results were low, suggesting a high level of clinical evidence. These results support the use of NOACs have similar safety and efficacy profile in the treatment of LVT.

Doubts on the efficacy of NOACs for the treatment of LVT exist, as there may be intrinsic mechanistic differences between LVT and thrombus associated with atrial fibrillation (33). However, thrombosis associated with endocardial changes in myocardial infarction should theoretically be transient and is different from that related to mechanical valves, in which case NOACs should be avoided (31). Thus, although current evidence suggests NOACs achieve similar clinical outcomes compared with VKAs for the treatment of LVT, further large-scale clinical trials are needed to establish more robust clinical evidence.

Concomitant antiplatelet therapy was broadly used in the included studies (65% ~ 92.1%); furthermore, 38%~69.3% of patients were prescribed with dual antiplatelet therapy for other indications (e.g., acute coronary syndrome, percutaneous coronary intervention) (11–13, 24). Co-prescribed antithrombotic therapy, notably triple antithrombotic strategy, undoubtedly increases the bleeding risk (11, 34); however, this can be alleviated by the novel antithrombotic strategy indicated in several recent researches, which revealed the superiority of the regimen including a NOAC and a P2Y_12_ inhibitor over traditional triple antithrombotic strategy (35, 36).

Today, we are facing two main dilemmas in the anticoagulation of ventricular thrombus. One is the choice of anticoagulation intensity. The therapeutic dose of NOACs for venous thromboembolism is higher than the prophylactic dose for stroke prevention in patients with non-valvular atrial fibrillation, making it difficult to extrapolate the appropriate therapeutic dose for LVT. Moreover, studies have shown that the use of warfarin to control the INR to 3-4 ameliorates the resolution of ventricular thrombus in patients who failed in NOACs therapy (23). Another dilemma is the duration of anticoagulation. It may be challenging to make the decision of anticoagulation discontinuation in cardiomyopathies (e.g., dilated cardiomyopathy) that cannot be fully recovered and often have no acute event time point.

### Limitations

Our meta-analysis has several limitations. Firstly, studies pooled in our research were retrospective studies with small sample sizes; further prospective, large-scale, randomized clinical trials are needed to establish more robust clinical evidence. Secondly, all six included studies used TTE rather than CMR as a primary diagnostic standard, which is likely to introduce misdiagnoses. Thirdly, although antiplatelet use was a significant confounder for the outcomes, we cannot fully adjust our results due to the lack of individual data.

## Conclusion

In this meta-analysis to investigate the differences between NOACs and VKAs for the treatment of LVT, no differences were found in the efficacy and safety, which inferred that NOACs might be a promising candidate for LVT therapy.

## Data Availability Statement

The original contributions presented in the study are included in the article/Supplementary Material, further inquiries can be directed to the corresponding authors.

## Author Contributions

HX: formal analysis, visualization, and writing-review & editing. Y-MC: investigation, data curation, and writing original draft. Y-LD: conceptualization, project administration, and methodology. JZ: validation and resources. Y-FJ: software. Y-FZ: supervision and funding acquisition. All authors contributed to the article and approved the submitted version.

## Conflict of Interest

The authors declare that the research was conducted in the absence of any commercial or financial relationships that could be construed as a potential conflict of interest.

## Supplementary Material

Supplementary Figure 1 Meta-analysis of vitamin K antagonists vs. novel oral anticoagulants for the endpoints of thromboembolic events after excluding 64 individuals who switched treatment. Tests for differences were based on T tests using fixed effect models.

Supplementary Figure 2 Contour-funnel plot in conjunction with the trim-and-fill method used to identify the causes of the observed asymmetry in the funnel plot for thromboembolic events.

Supplementary Table 1 Definitions for clinically significant bleedings in included studies.
