# Supplementary figures and images for: Safety and Efficacy of Vitamin K Antagonists vs. Novel Oral Anticoagulants in Patients With Left Ventricular Thrombus: A Meta-Analysis
Source: Front Cardiovasc Med. 2021 Apr 29;8:636491. doi: 10.3389/fcvm.2021.636491 (PMC8118127; doi:10.3389/fcvm.2021.636491)

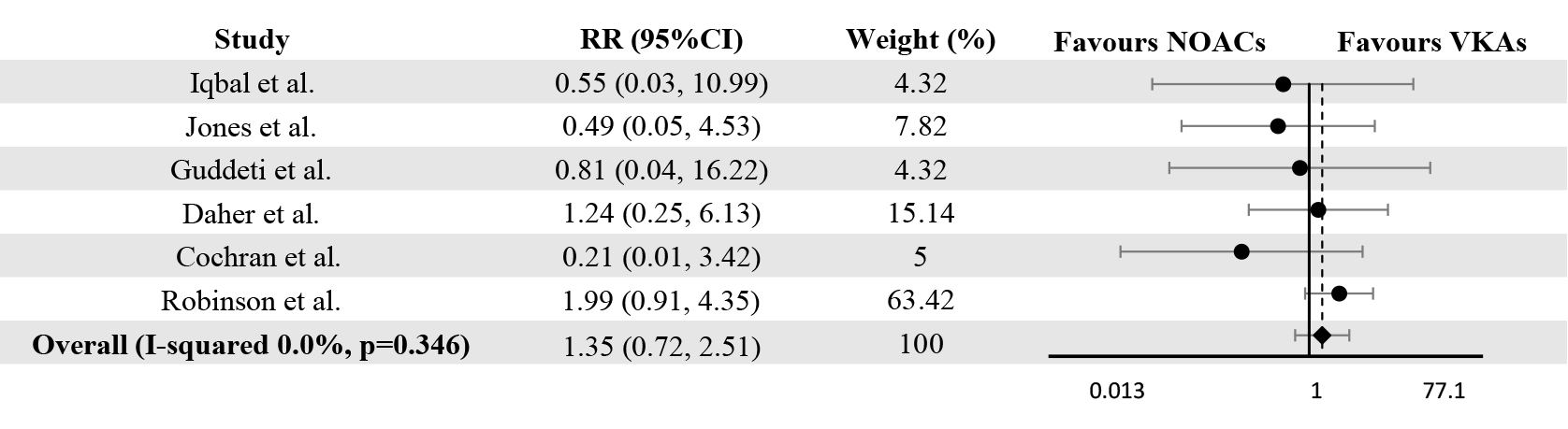

Supplement: Supplementary Figure 1 — Meta-analysis of vitamin K antagonists vs. novel oral anticoagulants for the endpoints of thromboembolic events after excluding 64 individuals who switched treatment. Tests for differences were based on T tests using fixed effect models. [file Image_1.TIF]

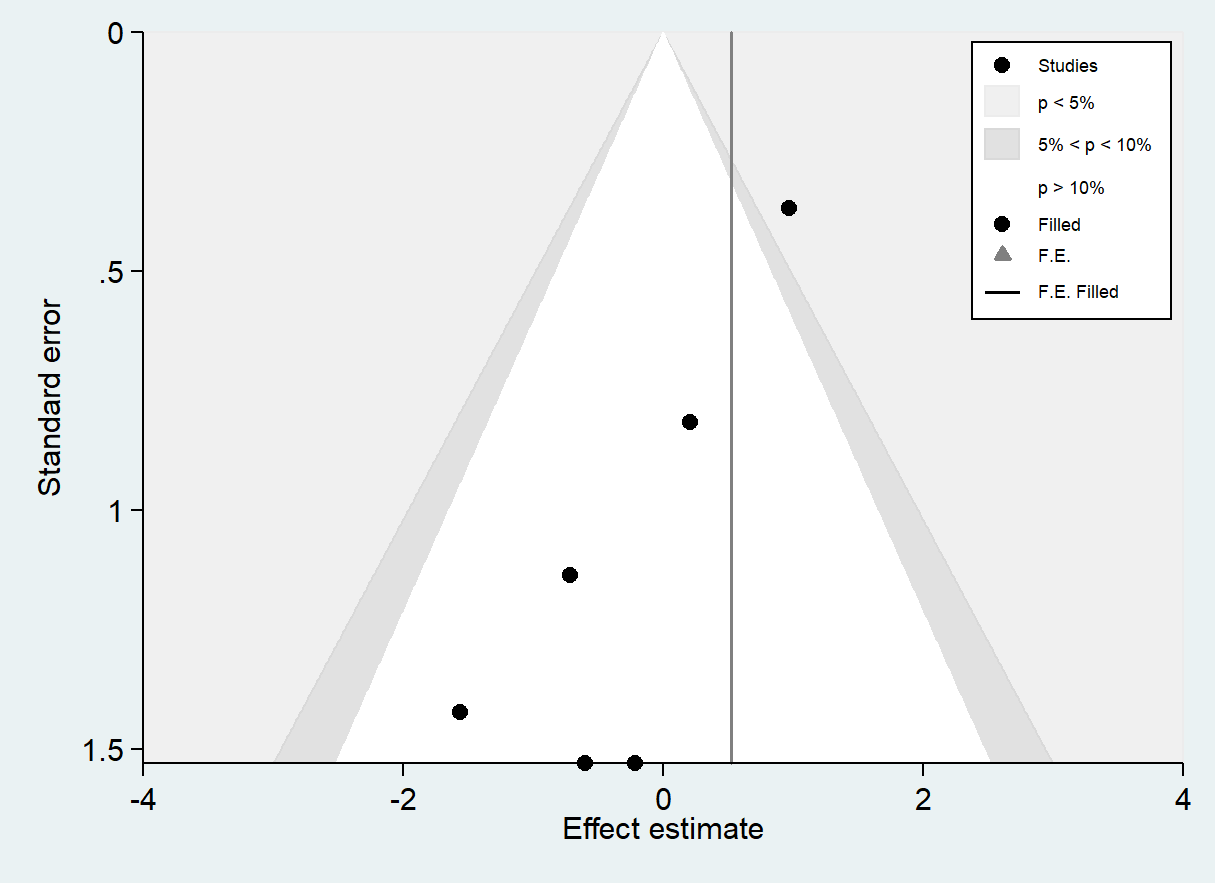

Supplement: Supplementary Figure 2 — Contour-funnel plot in conjunction with the trim-and-fill method used to identify the causes of the observed asymmetry in the funnel plot for thromboembolic events. [file Image_2.TIF]
