# Supplementary material for: Safety and Efficacy of Vitamin K Antagonists vs. Novel Oral Anticoagulants in Patients With Left Ventricular Thrombus: A Meta-Analysis
Source: Front Cardiovasc Med. 2021 Apr 29;8:636491. doi: 10.3389/fcvm.2021.636491 (PMC8118127; doi:10.3389/fcvm.2021.636491)
Supplement: Supplementary Table 1 — Definitions for clinically significant bleedings in included studies. [file Table_1.DOCX]

| Study | Country | Design | Definitions for clinically significant bleedings |
| --- | --- | --- | --- |
|  |  |  |  |
| Iqbal et al. | UK | retrospective | bleeding events requiring hospitalization |
| Jones et al. | UK | prospective | bleeding events (BARC ≥3) |
| Guddeti et al. | USA | retrospective | any life-threatening bleeding, drop in hemoglobin ≥2 grams or bleeding requiring hospitalization or evaluation by endoscopy |
| Daher et al. | France | retrospective | NR |
| Cochran et al. | USA | retrospective | TIMI bleeding criteria—minimal, minor, and major included |
| Robinson et al. | USA | retrospective | bleeding events requiring cessation in anticoagulation |

Supplement table 1. Definitions for clinically significant bleedings in included studies.

Abbreviations: BARC, Bleeding Academic Research Consortium; TIMI, thrombolysis in Myocardial Infarction.

NR refers to no available data.
